# Granulocyte colony-stimulating factor and reproductive medicine: A review

**Published:** 2015-04

**Authors:** Marcelo Borges Cavalcante, Fabrício DA Silva Costa, Ricardo Barini, Edward Araujo Júnior

**Affiliations:** 1 *Department of Gynecology and Obstetrics, University of Fortaleza, Fortaleza-CE, Brazil.*; 2 *Department of Perinatal Medicine, Pregnancy Research Centre, University of Melbourne, The Royal Women’s Hospital, Melbourne, Victoria, Australia.*; 3 *Department of Obstetrics and Gynaecology, Pregnancy Research Centre, University of Melbourne, The Royal Women’s Hospital, Melbourne, Victoria, Australia.*; 4 *Department of Obstetrics and Gynecology, State University of Campinas, Campinas-SP, Brazil.*; 5 *Department of Obstetrics, Paulista School of Medicine, São Paulo Federal University, São Paulo-SP, Brazil.*

**Keywords:** *Granulocyte colony*-*stimulating factor*, *Immunotherapy*, *Recurrent miscarriage*, *Reproductive medicine*, *Review*

## Abstract

**Background:**

Recently, the use of granulocyte colony-stimulating factor (G-CSF) has been proposed to improve pregnancy outcomes in reproductive medicine.

**Objective:**

A systematic review of the current use of G-CSF in patients who have difficulty conceiving and maintaining pregnancy was performed.

**Materials and Methods:**

Two electronic databases (PubMed/ Medline and Scopus) were searched. Study selection, data extraction and quality assessment were performed in duplicate. The subject codes used were granulocyte colony-stimulating factor, G-CSF, recurrent miscarriage, IVF failure, and endometrium.

**Results:**

The search of electronic databases resulted in 215 citations (PubMed/ Medline: 139 and Scopus: 76), of which 38 were present in both databases. Of the remaining 177 publications, seven studies were included in the present review.

**Conclusion:**

Treatment with G-CSF is a novel proposal for immune therapy in patients with recurrent miscarriage and implantation failure following cycles of IVF. However, a larger number of well-designed studies are required for this treatment to be established.

## Introduction

The World Health Organization (WHO) defines recurrent miscarriage (RM) as the occurrence of three or more consecutive miscarriages before 20 weeks of gestation (1). Recently, the American Society for Reproductive Medicine (ASRM) defined RM as two or more consecutive pregnancy losses documented by ultrasound or histopathologic examination (2). This condition affects approximately 2-4% of couples who are trying to have a baby. Genetic abnormalities in at least one member of the couple, hormonal changes, congenital uterine malformations, cervical incompetence, and infectious and environmental factors are responsible for approximately one-half of cases of RM. The causes of RM in the other one-half of cases remain unclear. In this context, immunological causes have generated considerable research interest because they can participate in the pathophysiology of pregnancy loss of unknown cause (3).

Recurrent implantation failure refers to a failure to achieve a clinical pregnancy after transfer of at least four good quality fresh or frozen embryos in a minimum of three cycles in a woman <40 years of age (4). Several other definitions have been reported; thus, it has been difficult to standardize a definition for better comprehension of this reproductive condition (5). The failure to implant may be a consequence of embryo or uterine factors (4). The pregnancy rate (PR) following cycles of in vitro fertilization (IVF) is correlated with endometrial thickness. A large number of studies have determined the minimal thickness to be approximately 7 mm (6). Despite the fact that thin endometrium is present in a small number of cases of implantation failure, there are no approved therapies for increasing endometrial thickness. Therapies that have already been tested include extended oestrogen administration and treatment with low-dose aspirin, vaginal sildenafil citrate, and a combination of pentoxifylline and tocopherol and gonadotropin- releasing hormone agonist (7-11). 

Granulocyte colony-stimulating factor (G- CSF) is a recently discovered cytokine. It was first recognized and purified in mice in 1983. The human form (hG-CSF) was cloned three years later in 1986 (12, 13). G-CSF is a hematopoietic lineage-specific cytokine produced by cells of the bone marrow, stromal cells, fibroblasts, endothelial cells, monocytes and macrophages. Its main function is to stimulate the proliferation and differentiation of neutrophils in the bone marrow and control their release to the bloodstream. In mature neutrophils, G-CSF works by increasing phagocytosis and the oxidative process (14).

The biological activities of hG-CSF are mediated by a specific receptor on the cell surface of responding cells. This receptor (G- CSF-R) is present on myeloid progenitor cells, myeloid leukaemia cells, mature neutrophils, platelets, monocytes, lymphoid cells and some T cells and B cells. In addition to these cells of hematopoietic lineage, receptors for G-CSF are found in several non- hematopoietic cell types, including endothelial cells, placenta cells, trophoblastic cells and granulosa luteinized cells. Studies in animals and humans have shown that G-CSF contributes to successful reproduction by enhancing embryo implantation and ovarian function, contributing to reduced pregnancy loss, promoting endometrial thickening and improving the pathophysiology of endometriosis (15, 16). 

The imbalance in the immune response due to T helper 1 and T helper 2 cell function, natural killer cell cytotoxicity, HLA compatibility and dysfunction of CD4^+^CD25^+^ T cells are some of the immunological mechanisms potentially responsible for failures in embryo implantation and consequently RM (17). The use of lymphocyte immunotherapy, intravenous human immunoglobulin, corticosteroids, lipid infusions, anti-TNF drugs and seminal plasma suppositories have been proposed as immunological treatments over the past decades (18-23). 

However, due to a lack of conclusive studies, there is still no consensus on which immunotherapy is indicated in cases of RM associated with alloimmune causes. Studies in rat models have demonstrated an anti- abortive effect of G-CSF; however, in a pharmacological study conducted in a rabbit model, administration of recombinant G-CSF (rG-CSF) was associated with a high rate of abortion (24, 25). Recent studies have proposed the use of G-CSF as immune therapy in cases of RM of unknown cause as well as in cases of implantation failure in women with thin endometrium. The possible mechanisms involved in improving pregnancy outcomes are not yet known. G-CSF administration appears to be associated with an increase in regulatory T cells and dendritic cells and appears to influence endometrial expression of genes crucial for the implantation process, including endometrial vascular remodelling, local immune modulation and cellular adhesion pathways (26, 27). 

The aim of the present review is to assess whether there are consistent data in the literature regarding the routine use of G-CSF in cases of RM of unknown cause and cases of implantation failure due to thin endometrium.

## Materials and methods

The present review was performed according to the PRISMA guidelines. A systematic search for studies investigating the use of G-CSF in cases of RM and IVF failure due to thin endometrium was performed. The search was included the PubMed/ Medline and Scopus databases from 1^st ^January 1980 to 30^th^ July 2014. The search was involved both subject codes and keyword searches. The subject codes used were granulocyte colony-stimulating factor, G-CSF, recurrent miscarriage, IVF failure, and endometrium.

Articles identified by the initial search were independently evaluated by two authors, according to the following inclusion criteria: 1) types of studies, 2) population, 3) intervention (use of G-CSF), and 4) outcomes (miscarriage, live birth rate, and endometrial thickness). Articles were limited to human studies published in English. All study designs (case reports, observational cohort studies, case- control studies, and randomized controlled trials) were included. Studies investigating the use of G-CSF in women with RM and implantation failure in IVF cycles due to thin endometrium were selected. Experimental studies and reviews on the subject were excluded. 

**Figure 1:**
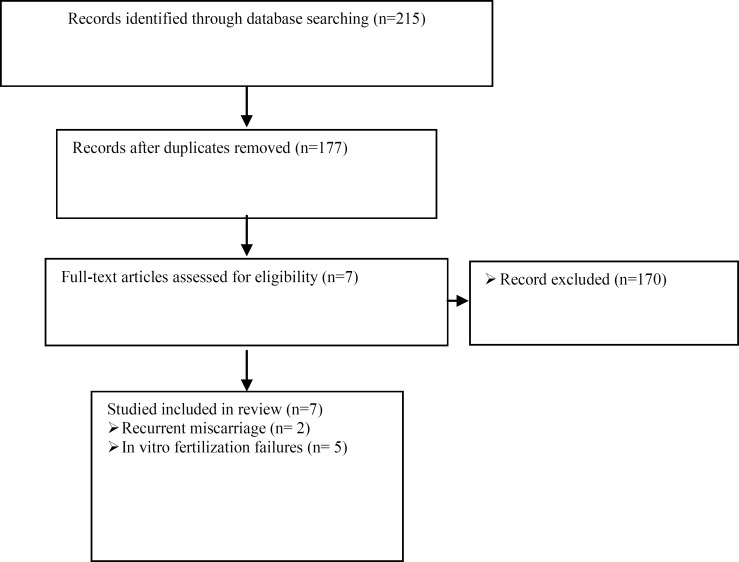
Flowchart outlining the database search

Routes of administration of G-CSF were assessed (intrauterine infusion in cases of implantation failure in IVF cycles and subcutaneous administration in cases of RM). Different treatment protocols between the studies were noted. Figure 1 presents a flowchart outlining the database search.

## Results

The electronic search resulted in 215 citations (PubMed/ Medline: 139, and Scopus: 76), of which 38 were present in both databases. Of the remaining 177 publications, seven studies were included in the present review. 

Two studies that investigated RM were included: one randomized controlled trial and one retrospective cohort study (Table I). Five publications that investigated IVF failures were included: one case series, three retrospective observational studies, and one randomized controlled trial (Table II).

**Table I:** Granulocyte colony-stimulating factor and recurrent miscarriage

**Study**	**Design**	**Participants**	**Intervention**	**Control**	**Outcomes**
Scarpelli & Sbracia (2009)	Randomized controlled trial	68 patients rG-CSF group: 35 Control group: 33	Filgastrim-rG-CSF 1 µg (100.000 IU)/kg/day from the 6^th ^day after ovulation until the occurrence of menstruation or to the end of the 9^th^ week of gestation	Saline solution	Miscarriages
Santjohanser et al (2013)	Retrospective cohort study	127 patients undergoing in vitro fertilization	G-CSF: 11 patients received 34×106 IU once per week and 38 patients received 13×106 IU twice per week starting on the day of embryo transfer until the 12^th^ week of gestation	Not treated or treated with other medications: enoxaparin 40 mg subcutaneously once per day, acetylsalicylic acid (100 mg/day), folic acid (5 mg/day) or prednisone/ dexamethasone (2.5-5.0 mg/0.5 mg/day) starting in the middle of the previous cycle until the evidence of an embryonic heart beat and doxycycline (100 mg/day for 5 days) beginning at the day of the embryo transfer.	Pregnancy rate Live birth rate

G-CSF: Granulocyte colony-stimulating factor.

**Table II:** Granulocyte colony-stimulating factor and in vitro fertilization failure

**Study**	**Design**	**Participants**	**Intervention**	**Control**	**Outcomes**
Gleicher et al (2011)	Case report	4	Intrauterine infusion of 30×106 IU (300 µg/1 mL) of rG-CSF performed for 6-12 hr before hCG injection	-	-
Gleicher et al (2013)	Prospective observational cohort	21 in vitro fertilization patients women with endometrium <7 mm on the day of hCG administration	Intrauterine infusion of 30×106 IU (300 µg/1 mL) of rG-CSF performed for 6-12 hr before hCG injection	Did not include a control group	Endometrial thickness
Barad et al (2014)	Prospective randomized cohort	141 normal in vitro fertilization patients (73 rG-CSF group and 68 placebo group)	Intrauterine infusion of 30×106 IU (300 µg/1 mL) of rG-CSF performed for 6-12 hr before hCG injection	Intrauterine infusion of saline solution	Endometrial thickness Implantation rate Clinical pregnancy rate
Li et al (2014)	Retrospective observational cohort	59 patients submitted to frozen embryo transfer (34 rG-CSF group and 25 no treated group)	Intrauterine infusion of 100 µg/0.6 mL of rG-CSF	No treatment	Implantation rate Clinical pregnancy rate
Kunicki et al (2014)	Retrospective observational cohort	37 in vitro fertilization patients with endometrium <7 mm on the day of hCG administration	Intrauterine infusion of 30×106 IU (300 µg/1 mL) of rG-CSF	Did not include a control group	Endometrial thickness

G-CSF: granulocyte colony-stimulating factor

hCG: human chorionic gonadotropin.

## Discussion

The use of rG-CSF as a treatment option for couples with RM was first proposed by Scarpellini and Sbracia (28). A total of 68 women with a history of RM of unknown cause who had been treated with intravenous human immunoglobulin were randomly assigned to undergo treatment with either rG-CSF or placebo. The treated group was consisted of 35 women who were received a dose of 1 µg (100,000 IU)/kg/day of Filgrastim (Neupogen, Dompe, Italy) subcutaneously from the sixth day after ovulation until the onset of menstruation or the end of the ninth week of pregnancy. The placebo group was consisted of 33 women who were received saline by the same route of administration and for the same time period as the treated group. All women in the study became pregnant spontaneously within three months (28).

The success rate of Scarpellini and Sbracia research was 82.8% in the treated group (29 live births in 35 pregnancies) and 48.5% in the placebo group (16 live births in 33 pregnancies). The difference between the groups was statistically significant (p=0.0061, OR=5.1; 95% CI: 1.5-18.4). The number of patients needed to treat (NNT) for one additional live birth was 2.9 (95% CI: 2.1-10.3). During pregnancy, the patients treated with rG-CSF also had higher levels of β-human chorionic gonadotropin (hCG) compared with those in pregnant women in the placebo group (28). In the group treated with rG-CSF, one case of skin rash and two cases of leukocytosis (white blood cell count >25,000 mL) were observed. In the placebo group, one case of gestational hypertension was observed. There was no difference in gestational age at abortion and birth weight between the two groups. No cases of congenital malformations were reported. Genetic testing was performed on the product of abortion in 14 of 23 miscarriages; chromosomal abnormalities were observed in one case in the treated group and two cases in the placebo group (28).

In 2013, Santjohanser *et al* evaluated the effect of G-CSF in patients with a history of RM (at least two early miscarriages) who underwent IVF (29). A total of 199 IVF cycles in 127 RM patients were studied. Three groups were compared: a group treated with G-CSF (49 patients), a group not treated with any medication (subgroup 1, 33 patients) and a group treated with other medications (subgroup 2, 45 patients). In the G-CSF group (n=49 patients), 11 patients received 34×10^6^ IU G-CSF once per week and 38 patients received 13×10^6^ IU G-CSF twice per week starting at the day of embryo transfer until the 12^th^ week of pregnancy. Subgroup 1 (n=33 patients and 46 cycles) did not receive any medication. Subgroup 2 was consisted of 45 patients (81 cycles) who were treated with other drugs: enoxaparin 40 mg subcutaneously once per day; acetylsalicylic acid (100 mg/day); folic acid (5 mg/day) or prednisone/dexamethasone (2.5-5.0 mg/ 0.5 mg/day), starting in the middle of the previous cycle until evidence of an embryonic heart beat was observed; and doxycycline (100 mg/day for five days) beginning on the day of the embryo transfer. All study patients received folic acid (0.5 mg) and progesterone vaginally (600 mg/day in the luteal phase until the 12^th^ week of pregnancy) (29). 

Santjohanser *et al *observed better reproductive results in G-CSF group (29). A PR of 47% and a live-birth rate (LBR) of 32% were achieved after G-CSF administration. In comparison with the G-CSF group, subgroup 2 (who received other medications) had a PR of 27% (p=0.016) and a LBR of 14% (p=0.006), while subgroup 1 (who received no medications) had a PR of 24% (p=0.016) and a LBR of 13% (p=0.016). two studies evaluating the use of G-CSF in recurrent miscarriage used different regimens (doses and frequency of administration). However, both showed a reduction in abortion rates (28, 29). Studies indicate improvement in PRs when G-CSF is administered in patients with thin endometrium at the time of embryo transfer (30). There are also data suggesting that G-CSF is involved in follicle development and may be a predictor of IVF outcome (31). However, situations in which there may be a real benefit in the use of G-CSF remain to be discovered.

In 2011 Gleicher *et al* described, for the first time, the use of rG-CSF for improvement of the endometrium in cases of women undergoing IVF with thin endometrium (30). At that time, four patients between 33 and 45 years of age were treated with intrauterine infusions of rG-CSF [30 MU (300 µg/1 mL)]. All patients became pregnant and had ongoing pregnancies, except for one patient who experienced an ectopic pregnancy. Given the promising initial results, Gleicher *et al* published an uncontrolled cohort study involving 21 patients in whom rG-CSF was administered by an intrauterine route to improve endometrial thickness (32). 

The mean age of the patients was 40.5±6.5 years and most had a diagnosis of reduced ovarian reserve [16/21 (76.2%)]. The cases described were the first performed in the researcher centre; the patients had a history of previous unsuccessful IVF attempts, averaging 2.0±2.1 (0-9) cycles. They also had a history of previous cancellation of attempts due to thin endometrium [0.1±0.4 (0-1) cycles]. The diagnosis of thin endometrium was performed on the day of hCG administration. Patients assigned to receive 30 MU (300 µg/mL) of rG-CSF had an endometrial thickness of ˂7 mm. The intrauterine infusion was performed for 6h/12h before hCG injection. The assessment of endometrial thickness was again performed on the day of follicular aspiration approximately 48 h after administration of rG-CSF. An additional intrauterine infusion of rG-CSF was administered in three of 21 cases (14.3%) in whom the endometrial thickness was still <7 mm. 

Among the 21 patients treated, there was a significant increase in endometrial thickness of 2.9±1.9 mm from the first infusion of rG-CSF to the embryo transfer procedure. The improvement in endometrial thickness alone also occurred when comparing the groups of patients who became pregnant [4/21 (19.0%)] and those who did not become pregnant [(17/21 (81.0%)]. In this small cohort study the effect of G-CSF in the improvement of endometrial thickness was observed. However, it is not possible to assess the impact of G-CSF use in improving pregnancy rate due to lack of a control group.

Kunicki *et al* evaluated pregnancy outcomes in a group of 37 patients with thin endometrium (<7 mm) who were subjected to new IVF being treated with rG-CSF, similar to the protocol described by Gleicher *et al* (32, 33). The PR in this cohort was 18.9% (7/37). The endometrial thickness improved significantly after a 72 hr infusion of rG-CSF in both groups (women who became pregnant and those who did not). However, there was no difference in endometrial thickness either before or after the infusion of rG-CSF, between women who became pregnant and those who did not. The use of rG-CSF in cycles of frozen embryo transfer (FET) with non-responsive (<7 mm) endometrium has been reported by Li *et al* (34). This study retrospectively analysed 59 patients who were divided into two groups, which were either treated (n=34) or not treated (n=25) with uterine infusions of 100 μg (0.6 mL) rG-CSF during endometrial preparation for FET. No significant differences between groups were observed with regard to the implantation rate and PR. In the treated group, there was no increase in endometrial thickness before uterine infusion of rG-CSF compared with that after infusion. 

Recently, Barad *et al* evaluated the effect of rG-CSF on the results of IVF cycles in women with normal endometrium thickness (35). A total of 141 women were included; 129 underwent cycles of ‘fresh’ IVF and 12 underwent FET. Patients were randomly assigned to two groups: 73 patients received an intrauterine infusion of rG-CSF 30 MU (300 µg/1 mL) and 68 patients received an intrauterine infusion of saline (control group). The intervention was performed on the morning of the administration of hCG. Barad *et al* concluded that the use of rG-CSF did not improve rates of implantation and pregnancy in this group of patients with normal endometrial thickness (35).

## Conclusion

The involvement of immunological factors in cases of RM of unknown causes and in cases of implantation failure seems to be a reality. However, the lack of scientific evidence supporting proposed immunotherapies raises questions about the true importance of immunological evaluation in these patients. Lymphocyte immunization, human intravenous immunoglobulin, infusion of lipids, anti-TNF drugs and steroids are examples of immunotherapies that have been proposed but are no longer supported. Why is there a lack of scientific evidence for these immunological treatments? 

The small number of controlled clinical studies, the heterogeneity of the study groups and the lack of selection criteria based on immunological parameters are some of the reasons for the lack of clarity in the medical literature. Treatment with G-CSF is a novel proposal for immune therapy in cases of RM and implantation failure in IVF cycles. To date, few studies have been conducted, and there are still many questions to be answered. Which group of patients will truly benefit from the treatment? What is the optimal dose and cycle period in which treatment should be initiated? What is the best route of administration (systemic or intrauterine)? Well-designed clinical studies should be conducted to provide answers to these questions.

## Conflict of interest

The authors declare no conflict of interest. 
